# The Week's Work

**Published:** 1911-08-12

**Authors:** 


### The Week's Work.

### THE BRITISH HOSPITALS ASSOCIATION.

In accordance with a suggestion made by Mr. Lloyd George to the deputation who had an interview with the Chancellor of the Exchequer on the 25th ult. in reference to the probable effects of the National Insurance Bill upon the voluntary hospitals, a meeting of the Special Committee of the British Hospitals Association conferred with the British Medical Association on the 4th inst. A general conversation took place, and as an outcome of the meeting, a joint committee, consisting of four members of each of the two Associations, was formed to consider further the various points which arose and to report thereon to their respective Associations. No definite resolutions were passed and the proceedings were private, but we understand that it was generally agreed that the effect of the National Insurance Bill in its original form must be most prejudicial to the voluntary hospitals, and that it must indeed tend to destroy their voluntary character.

### A NEW OPENING FOR DISPENSERS.

The list of medicines which merchant ships are required by the Board of Trade to carry is somewhat out of date, and, at the request of the General Shipowners' Society, the Board appointed a committee a short time ago to inquire into the matter and report. The Committee, which was composed of five medical men, representing the Board of Trade, the Liverpool shipowners, the Royal College of Physicians, the Royal College of Surgeons, and Mr. A. J. Phillips, representing the Pharmaceutical Society, has now finished its work. The most important of its recommendations is that on certain ships a qualified dispenser should be taken. The list of medicines to be carried has been thoroughly revised, one important innovation being the admission of compressed drugs. It is to be feared that in the past ship's captains have not always kept their medical stores properly replenished, and it is to be hoped that if the recommendations are adopted steps will be taken to ensure that the new regulations are observed. It is important that provision should be made for supplying the medical requirements of those who go down to the sea in ships. It is, we think, even more important that a qualified and experienced dispenser should be carried.

### THE MASTER OF GRESSENHALL WORKHOUSE.

At a recent sitting of the House of Commons, Mr. Boyle, the M.P. for Mid-Norfolk, strongly criticised the action of the Local Government Board in dismissing the Master of the Gressenhall Workhouse. The facts of the case are these: An old man, seventy-two years of age and destitute, tried to commit suicide by cutting his throat. Two constables discovered him, one of whom administered first aid while the other went for a doctor, who found it necessary to put ten stitches into the wound. For further treatment it seemed necessary that the patient should be taken to the infirmary, which was five miles off. The overseer of the poor having given an order accordingly, the man was conveyed thither, accompanied by the two constables, his son, and the clergyman of the parish. The infirmary was reached at two o'clock in the morning, and the porter, when aroused, assured the patient's friends that he did not think the man would be admitted. The master, who came down after some delay, confirmed the porter's statement. He argued that the would-be suicide must be a lunatic, and that the guardians had decided not to admit lunatics. The unfortunate old man had therefore to be taken back to the village whence he came, where, it was admitted, he was kindly treated and looked after by the policemen. On hearing of the case, the President of the Local Government Board felt it his duty to ask the master of the workhouse to resign, wisely judging that a man who came to such a decision in a case of emergency must be either too stupid or too callous for the post he filled. It is true that the pitying judgment of our courts of law says of anyone who has taken his own life under the strain of poverty and distress that he committed suicide while of unsound mind. But who would ever think of this as an argument for refusing needed help to the poor and distressed one who has not succeeded in cutting himself adrift from life's tangled coil? The master of Gressenhall, who reasons thus with such logical inhumanity must surely be a descendant of the great Bumble—one who has never learned that the letter killeth, while the spirit maketh alive. His forced resignation may be hard on him—though, as he and his wife are to receive their due pensions, they are not so much to be pitied; but it is not by any means so hard as he evidently had the will—and, unfortunately, also the power—to be to one poor soul at least who came under his control. We congratulate Mr. Burns on his decision, and even if, as is threatened, he is burned in effigy in the market-place of East Dereham, we have no doubt that he will regard the bonfire as a *feu de joie*.

### A MILLIONAIRE'S EFFORTS TO IMPROVE THE WORLD

One of the most interesting wills of the past year or two is that of Mr. Marino Corgialegno, a rich Greek merchant, one of the leading members of the Greek community in London. The "unloading" of our modern millionaires has often produced ironic laughter, not only among economists. This present instance, however, induces less bitter emotions. He is said to have left estate valued at £585,306 gross, and his first bequest of a public character is one of £2,000 to the Lord Mayor of London to be distributed by him among hospitals and charitable institutions. Subject to his wife's life interest, he left all his other property, amounting to nearly £500,000, upon trust for public uses, most prominent among which is £80,000 to found a new hospital in Athens. Although not of institutional interest, we cannot forbear from quoting the following bequests: £50,000 (on the suggestion of King George of Greece), as to £10,000 for barracks for the improved accommodation of the Greek Army, and as to £40,000 to be used in the uncontrolled discretion of King George of Greece for the improvement of the Greek Navy; and, still more interesting, £40,000 to institute a school at Athens on the lines of Eton or Harrow. Besides this, £25,000 is to be left upon trust to build a model prison in Greece; while, to return to our own interests, £25,000 is for a children's hospital at Athens. Other bequests are: £16,000 for draining the marshes at the end of the Gulf of Cutavos; £10,000 each for a polyclinical hospital in Athens and for the Hospital "Evangelismos." The residue of his property, which will amount apparently to not less than £40,000, he left for the improvement and embellishment of the city of Athens.

### THE "EYE WITNESS," THE DOCTORS, AND THE BILL.

It is interesting to note the comments of our youngest contemporary, the *Eye Witness*, certainly the most informative and at present the best written of the new weeklies, on the Insurance Bill as it affects the voluntary hospitals staff: "The really serious part of the resistance to it (the Insurance Bill) proceeds from the doctors. Everyone takes it for granted that the great trade-unions will surrender at discretion and allow themselves to be destroyed . . . but resistance from a profession which includes many wealthy men is another matter, and their claims are immediately admitted. Not that the doctors have not been perfectly right; they have. The Bill was copied in such a desperate hurry from Germany, and its finance was so ill-thought out, that the doctors would have had a very real grievance under it, and may still have. But it is significant that theirs is the only form of resistance which has been taken seriously." Our only comment on this is that in comparison with the numbers and average poverty of the profession the wealthy men in it are quite curiously few.

### CHANGES AT CHARING CROSS HOSPITAL.

The students of Charing Cross Hospital Medical School will, in future, take out their courses for the first and second professional examinations at King's College. This important alteration has recently been decided upon by the School Committee of Charing Cross Hospital after very serious discussion and the new arrangement comes into force at the beginning of the next winter session. For some time past the students of St. George's and Westminster Hospitals have been attending King's College for the first part of their curriculum, and we understand that the scheme has worked well. Greater facilities for practical work are available at the larger institution and the association with men from the other hospital has fostered a keen spirit of competition distinctly advantageous to the students. In the medical school of Charing Cross Hospital it is proposed to utilise the very valuable space which will now be set free in extending the accommodation for the pathological and bacteriological departments, in both of which spheres changes and improvements have lately been made. The lecturer on anatomy at Charing Cross, Dr. Alexander MacPhail, is also moving on to King's College to take up the lectureship there in that subject. Among other changes we may refer to the appointment of Dr. William Hunter as the new Dean of the school and the coming installation of Dr. W. W. C. Topley as bacteriologist and clinical pathologist.

### CHANGE OF SITE FOR BOURNEMOUTH SANATORIUM.

We announced on June 24 (page 315) that the Royal National Sanatorium for Consumption had decided to move from Bournemouth. We are now in a position to add that of all the sites recommended by the Committee of Management, which has been much indebted to the care and trouble of Dr. Davison, their senior visiting physician, the one most likely to be accepted is near Broadstone, in East Dorset. The owner, we are informed, is prepared to sell one hundred acres, or fifty with the option of doubling them, at £60 an acre, if not at a little less. Luckily the value of the present site of nearly three acres is great, and £500 has been promised from the Earl of Eldon. We are glad to note that Mr. W. F. D. Smith has recommended the adoption of the graduated labour system which has produced such good results at Frimley under Dr. Marcus Paterson. An architect "of considerable experience in sanatorium construction" is understood to have estimated the cost of a new institution of one hundred beds at £15,000, and one of two hundred beds at £20,000. Who is he?

### A NOTE ON MR. GRIFFITHS.

As Mr. Arthur Griffiths, whose very informing article upon the way in which the East Suffolk and Ipswich Hospital, of which he is secretary, adopted the uniform system of hospital accounts we publish elsewhere in this issue, is modest enough to confess his official experience of hospital work to be a short one, his previous record deserves attention. For some ten years he was accountant to the Felixstowe and Walton Urban District Council, and on his marriage in 1905 a roll-top desk and inkstand were presented to him. At the presentation the chairman of the council, Mr. D. J. Cowles, said that "four consecutive chairmen and several old members joined in expressing their good wishes." He acted also as deputy Town Clerk from 1907-10. On his retiring in April 1910 to take up his appointment as secretary to the Ipswich Hospital, Mr. Cowles read a testimonial which included the following: "Mr. Griffiths has been in the service of the local authority and district for the past eighteen years, and during the early part of that period he was chief assistant and cashier to the late clerk and solicitor to the Local Board. . . . His duties have included the negotiation and raising of loans, the preparation of statistics for and giving evidence at Local Government Board inquiries, the preparation of all rate estimates, and the drafting of reports and statistics on special subjects. . . . He has considerable knowledge of Local Government law and procedure, gained from very practical experience, and especially of the part which affects accounts and rating." Mr. Griffiths received at the same time an address from the Council's workmen. He has had special success with them at Ipswich for the hospital, and has found a new source of income in collecting from visitors to patients. It will be seen, in short, that he is entitled to a hearing, and the actual process of carrying through the change to the uniform system has been helped, no doubt, by his previous experience as an accountant.

### THIS WEEK'S DRUG MARKET.

Business in the drug market has naturally been quiet during the past week, owing to the holiday feeling which is prevalent everywhere. Nevertheless, several articles continue to attract attention. Opium still tends dearer and makers of codeine have advanced their prices by 1s. 8d. per ounce, while morphine is 1s. 6d. per ounce higher in price. Carbolic acid is again somewhat dearer. Oil of cloves and oil of star aniseed are also rather dearer. Up to the present holders of cod-liver oil have made no reduction in their quotations, but lower prices are expected before long. Essence of lemon is again rather dearer.

### NEW MATRON FOR OLDHAM ROYAL INFIRMARY

We understand that the Board of Governors of the Oldham Royal Infirmary has appointed Miss Lilian E. Davies (of the London Hospital) to be matron.

